# Neuropilin‐1 is required for endothelial cell adhesion to soluble vascular endothelial growth factor receptor 1

**DOI:** 10.1111/febs.16119

**Published:** 2021-08-27

**Authors:** Gianni Colotti, Cristina Maria Failla, Pedro Miguel Lacal, Mariangela Ungarelli, Federica Ruffini, Patrizio Di Micco, Angela Orecchia, Veronica Morea

**Affiliations:** ^1^ Institute of Molecular Biology and Pathology (IBPM) of the National Research Council (CNR) Rome Italy; ^2^ Laboratory of Experimental Immunology IDI‐IRCCS Rome Italy; ^3^ Laboratory of Molecular Oncology IDI‐IRCCS Rome Italy; ^4^ Laboratory of Molecular and Cell Biology IDI‐IRCCS Rome Italy; ^5^ Department of Biochemical Sciences ‘A. Rossi Fanelli’ Sapienza’ University of Rome Italy; ^6^ Present address: Department of Data Science The Institute of Cancer Research London UK

**Keywords:** angiogenesis, neuropilin‐1, peptides, vascular endothelial growth factor receptor 1, α5β1 integrin

## Abstract

Neuropilin‐1 (NRP‐1) is a semaphorin receptor involved in neuron guidance, and a co‐receptor for selected isoforms of the vascular endothelial growth factor (VEGF) family. NRP‐1 binding to several VEGF‐A isoforms promotes growth factor interaction with VEGF receptor (VEGFR)‐2, increasing receptor phosphorylation. Additionally, NRP‐1 directly interacts with VEGFR‐1, but this interaction competes with NRP‐1 binding to VEGF‐A165 and does not enhance VEGFR‐1 activation. In this work, we investigated in detail the role of NRP‐1 interaction with the soluble isoform of VEGFR‐1 (sVEGFR‐1) in angiogenesis. sVEGFR‐1 acts both as a decoy receptor for VEGFs and as an extracellular matrix protein directly binding to α5β1 integrin on endothelial cells. By combining cell adhesion assays and surface plasmon resonance experiments on purified proteins, we found that sVEGFR‐1/NRP‐1 interaction is required both for α5β1 integrin binding to sVEGFR‐1 and for endothelial cell adhesion to a sVEGFR‐1‐containing matrix. We also found that a previously reported anti‐angiogenic peptide (Flt_2‐11_), which maps in the second VEGFR‐1 Ig‐like domain, specifically binds NRP‐1 and inhibits NRP‐1/sVEGFR‐1 interaction, a process that likely contributes to its anti‐angiogenic activity. In view of potential translational applications, we developed a five‐residue‐long peptide, derived from Flt_2‐11_, which has the same ability as the parent Flt_2‐11_ peptide to inhibit cell adhesion to, and migration towards, sVEGFR‐1. Therefore, the Flt_2‐5_ peptide represents a potential anti‐angiogenic compound *per se*, as well as an attractive lead for the development of novel angiogenesis inhibitors acting with a different mechanism with respect to currently used therapeutics, which interfere with VEGF‐A165 binding.

## Graphical abstract

Endothelial cell adhesion to sVEGFR‐1, which is mediated by the α5β1 integrin/sVEGFR‐1 interaction, requires neuropilin‐1. Neuropilin‐1 interacts with both α5β1 integrin (on the same cell membrane) and sVEGFR‐1 (in the extracellular matrix) with high affinity, likely stabilizing the α5β1 integrin/sVEGFR‐1 interaction. Peptides Flt_2‐11_, Flt_2‐8_ and Flt_2‐5_, which are able to inhibit neuropilin‐1/sVEGFR‐1 interaction, have anti‐angiogenic activity that might be exploited for therapeutic purposes.

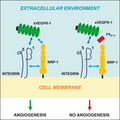

**Abbreviations**
3D: three‐dimensional
ECs: endothelial cells
NIP: neuropilin‐interacting protein
NRP: neuropilin
PDB: Protein Data Bank
RU: resonance units
SASA: solvent accessible surface area
shRNA: small hairpin RNA
SPR: surface plasmon resonance
sVEGFR‐1: soluble VEGFR‐1
tmVEGFR‐1: transmembrane VEGFR‐1
VEGF: vascular endothelial growth factor
VEGFR: VEGF receptor

## Introduction

Neuropilin‐1 (NRP‐1) is a transmembrane protein involved in diverse cellular functions [1]. It is the receptor of different members of the semaphorin family implicated in axon guidance in neurons [2, 3]; it acts as a co‐receptor for vascular endothelial growth factor (VEGF) family members in endothelial cells (ECs) [4, 5], and it mediates cell adhesion to the extracellular matrix [6]. Moreover, it has been shown that NRP‐1 stimulates growth of different tumour types [7, 8, 9], while NRP‐1 silencing suppresses cancer cell growth [10]. Further, NRP‐1 has been proposed to modulate immune cell activities in tumour immune surveillance [11]. Importantly, NRP‐1 has been recently demonstrated to be a host factor for SARS‐CoV‐2 infection [12, 13].

Neuropilin‐1 structure comprises three regions: extracellular, membrane and cytoplasmic. The extracellular region, in turn, comprises five domains: (1) two complement‐binding like domains (a1 and a2); (2) two coagulation factor V/VIII homology domains (b1 and b2); and (3) one MAM domain homologous to metalloendopeptidase meprin, A5 and mu‐phosphatase (c). Some of these regions have been associated with specific NRP‐1 functions. The a1, a2 and b1 domains have been shown to be involved in class 3 semaphorin binding. The b1‐b2 domains are implicated in both VEGF binding [14] and cell adhesion [1]. The role of the c domain is less clear. It was initially proposed to be involved in dimerization [2], based on cell experiments on NRP‐1 deletion variants and in analogy with other MAM domains. This hypothesis was subsequently challenged by the results of isothermal titration calorimetry experiments on the purified MAM domain, which indicate that, even if homo‐dimerization took place, the association constant would be weak [15]. Other roles proposed for this domain include the separation of the a1a2b1b2 domains from the membrane and their correct positioning for other intermolecular interactions [15]. The intracytoplasmic tail comprises ~ 40 amino acids and interacts with the neuropilin‐interacting protein (NIP) [16].

Interaction with NRP‐1 mediates, at least in part, cell signalling by growth factors belonging to the VEGF family, including VEGF‐A isoforms VEGF‐A165, VEGF‐A189 and VEGF‐A206 [17, 18, 19] but not VEGF‐A145 [20]. When co‐expressed with the VEGF receptor (VEGFR)‐2, NRP‐1 enhances VEGF‐A165 binding to VEGFR‐2 and VEGF‐A165‐mediated chemotaxis [5]. Additionally, NRP‐1 functions as an enhancer of VEGF‐A165‐mediated mitogenic response in human ECs [21]. Consistent with NRP‐1 role in modulating VEGF‐A165 availability and functions, NRP‐1 knock‐out mice die at the embryo stage with defects in both the nervous and cardiovascular systems [22], whereas NRP‐1 over expression causes anomalies at the cardiovascular level, such as excess capillaries and blood vessels, and abnormal heart [23].

Neuropilin‐1 has additional effects on ECs, which are not exerted by interference with VEGF‐A165 signalling. NRP‐1 regulates EC adhesion to extracellular matrix proteins independent of VEGFR‐2, as demonstrated by using RNA interference‐mediated silencing of NRP‐1 or VEGFR‐2 in primary human ECs [21]. Moreover, neither class 3 semaphorins or VEGF‐A165 interfere with NRP‐1 action in cell adhesion [1]. NRP‐1 also interacts with α5β1 integrin at sites of EC adhesion to fibronectin [24] and with β1 integrin subunit in tumour cells [25]. The interaction with α5β1 integrin in ECs is mediated by the extracellular region, and regulation of α5β1 integrin binding to fibronectin results from the interaction of the cytoplasmic tail with NIP and subsequent activation of integrin internalization and vesicle motility. On these bases, regulation of α5β1 integrin trafficking was proposed to be one of the mechanisms by which NRP‐1 exerts its role in angiogenesis [24].

Neuropilin‐1 was also shown to directly and reversibly interact with VEGFR‐1 [26, 27]. This interaction competed with NRP‐1 binding to VEGF‐A165 and did not enhance VEGFR‐1 activation or downstream signalling. The biological significance of this interaction is still unclear.

In addition to VEGFR‐1 transmembrane form (tmVEGFR‐1), soluble isoforms of the receptor are originated by alternative splicing of the same gene [28, 29, 30]. VEGFR‐1 soluble isoform produced by ECs (sVEGFR‐1) comprises the 656 N‐terminal residues in the extracellular region (encompassing the first six immunoglobulin (Ig)‐like domains), and a specific C‐terminal sequence of 30 amino acids. sVEGFR‐1 has a dual role in angiogenesis: (a) On the one hand, sVEGFR‐1 binds VEGF‐A165. This results in a reduced amount of VEGF‐A165 available for VEGFR‐2 binding and, therefore, in a downregulation of VEGFR‐2‐mediated angiogenic signalling. (b) On the other hand, sVEGFR‐1 is deposited by ECs in the extracellular matrix. Here, sVEGFR‐1 is involved in cell adhesion and migration via a direct interaction with α5β1 integrin [31], through which it triggers molecular signals of motility and angiogenesis [32]. Moreover, sVEGFR‐1/β1 integrin interaction has been demonstrated to be involved in the progression and response to anti‐angiogenic therapies in squamous cell lung carcinoma [33].

With the goal to identify the sVEGFR‐1 region interacting with α5β1 integrin, we previously designed and tested twelve peptides, which cover almost entirely the surface of the second extracellular Ig‐like domain of VEGFR‐1 [34]. One of these peptides, comprising the 11 amino acid residue sequence NITVTLKKFPL [34] had been previously reported with the name Flt_2‐11_ by a different group [35]. They found that Flt_2‐11_ had anti‐angiogenic activity *in vivo*, but was not able to either bind VEGF‐A165 or inhibit VEGF‐A165 interaction with ECs [35]. Our subsequent studies showed that peptide Flt_2‐11_ (a) specifically inhibited EC adhesion to sVEGFR‐1 but not to fibronectin [34]; (b) did not directly support α5β1 integrin‐mediated EC adhesion [34] and (c) inhibited EC migration towards sVEGFR‐1 [36].

In the present work, we report that peptide Flt_2‐11_ binds to NRP‐1 and inhibits NRP‐1 interaction with sVEGFR‐1. Hence, we take advantage of the peptide mechanism of action to elucidate the role of the NRP‐1/sVEGFR‐1 interaction in angiogenesis. Finally, we develop a five‐residue derivative of peptide Flt_2‐11_ that has the same anti‐angiogenic properties as the parent Flt_2‐11_.

These results expand our knowledge about NRP‐1 role in angiogenesis and indicate that NRP‐1 interaction with VEGFR‐1 has a role in the modulation of EC adhesion to the extracellular matrix. Taken together, the discovery of a molecular mechanism involved in angiogenesis that is independent of VEGF‐A165 binding, and of a short peptide able to inhibit this mechanism, opens the road to the development of novel anti‐angiogenic therapies, both alternative and complementary to those targeting VEGF‐A165 interactions.

## Results

### Flt_2‐11_ peptide binds to NRP‐1 and specifically inhibits NRP‐1 interaction with VEGFR‐1

**Fig. 1:**
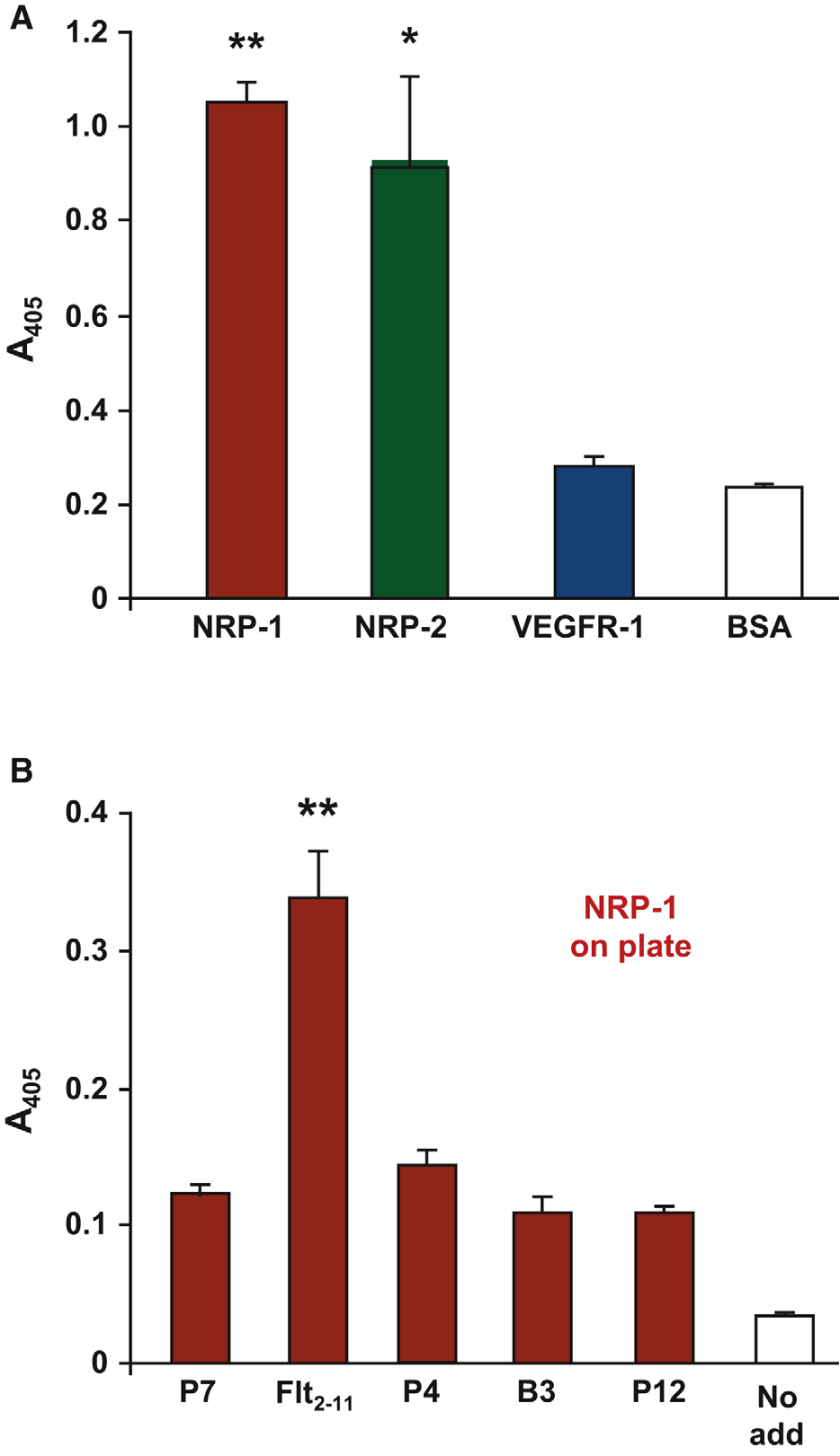
Flt_2‐11_ peptide binds to NRP‐1. (A) Solid‐phase binding assay of biotinylated Flt_2‐11_ peptide on a plate coated with NRP‐1, NRP‐2, VEGFR‐1 and BSA, as a negative control. (B) Solid‐phase binding assay in the presence of different biotinylated peptides (P7, Flt_2‐11_, P4, P12 and B3) derived from the second Ig‐like domain of VEGFR‐1 [34, 36], or in peptide absence (No add), on NRP‐1‐coated plates. Attached peptides were quantified by incubation with alkaline phosphatase‐conjugated streptavidin and a colorimetric assay. Experiments were performed in triplicate and repeated at least three times, with comparable results. Representative experiments are shown. Data are reported as mean ± SE of peptide adhesion with respect to control (peptide absence). Student’s *t*‐test: *P ≤ 0.05; **P ≤ 0.01.

To elucidate the mechanism by which the anti‐angiogenic Flt_2‐11_ peptide inhibits EC adhesion to, and migration towards, sVEGFR‐1, we first investigated the peptide ability to bind known VEGFR‐1 interactors. As shown in Fig. 1A, biotinylated Flt_2‐11_ peptide was able to bind NRP‐1, whereas it did not bind to VEGFR‐1 itself. Flt_2‐11_ peptide also bound neuropilin‐2 (NRP‐2), even though a direct interaction between NRP‐2 and VEGFR‐1 has not been previously demonstrated [37]. Additionally, Flt_2‐11_ peptide had been reported not to bind either VEGF‐A165 or α5β1 integrin [34, 36].

**Fig. 2:**
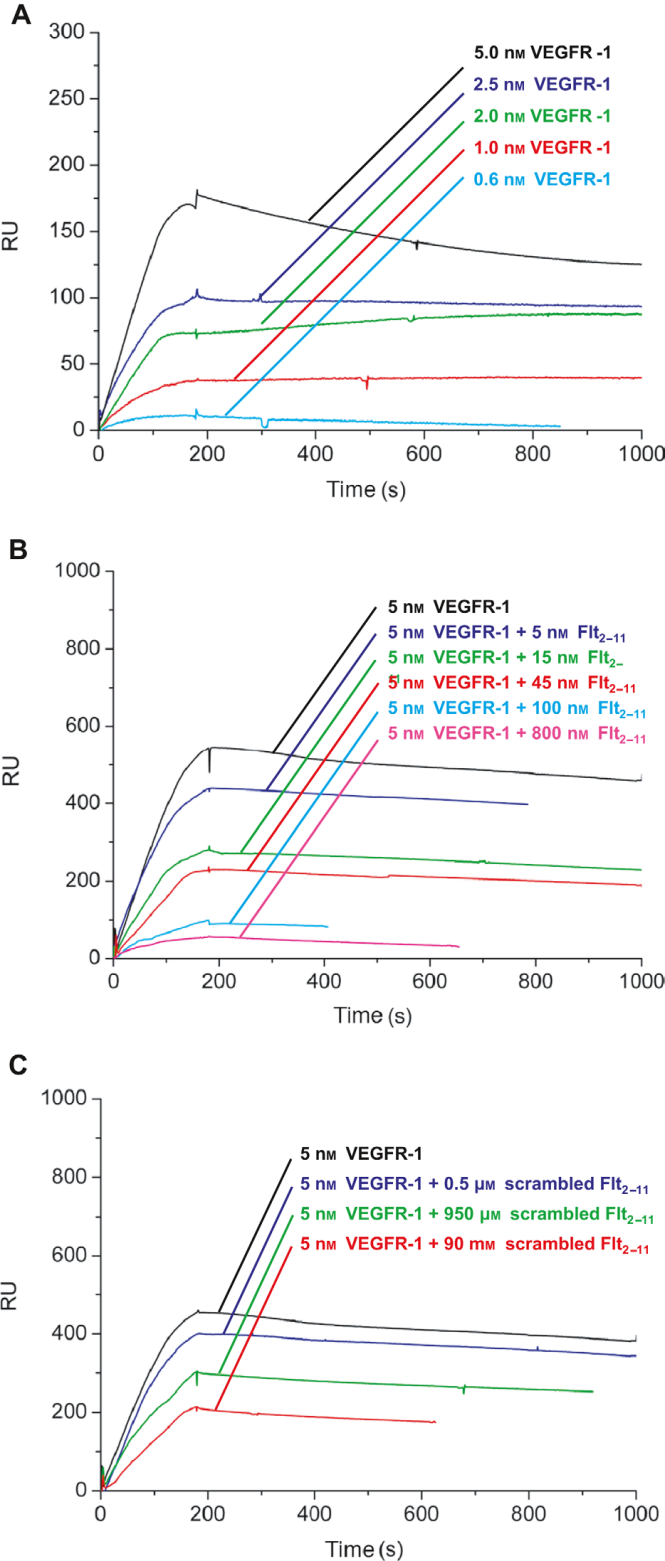
Flt_2‐11_ peptide inhibits NRP‐1 binding to VEGFR‐1. SPR analysis of the interaction between VEGFR‐1 and NRP‐1 in peptide absence (A), or presence of Flt_2‐11_ peptide (B) or scrambled Flt_2‐11_ peptide (C). NRP‐1 is immobilized on the sensor chip. (A) VEGFR‐1 is injected at different concentrations (from bottom to top: 0.6 nm, cyan; 1 nm, red; 2 nm, green; 2.5 nm, blue; and 5 nm, black). Association and dissociation kinetic constants are *k*_on_ = 1.2 × 10^4^ M^−1^·s^−1^ and *k*_off_ = 3 × 10^−4^ s^−1^; the thermodynamic dissociation constant is *K*_D_ = 25 ± 4 nm. (B) VEGFR‐1 is injected at 5 nm together with peptide Flt_2‐11_ at different concentrations (from top to bottom: 0, black; 5 nm, blue; 15 nm, green; 45 nm, red; 100 nm, cyan; and 800 nm, magenta). Flt_2‐11_ peptide effectively inhibits NRP‐1/VEGFR‐1 interaction (*K*_i_ = 20 nm). (C) VEGFR‐1 is injected at 5 nm together with ‘scrambled’ Flt_2‐11_ peptide at different concentrations (from top to bottom: 0, black; 0.5 μm, blue; 950 μm, geen; and 90 mm, red). Scrambled Flt_2‐11_ inhibits NRP‐1/VEGFR‐1 interaction only at high concentrations (*K*_i_ = 10 mm).

**Fig. 3:**
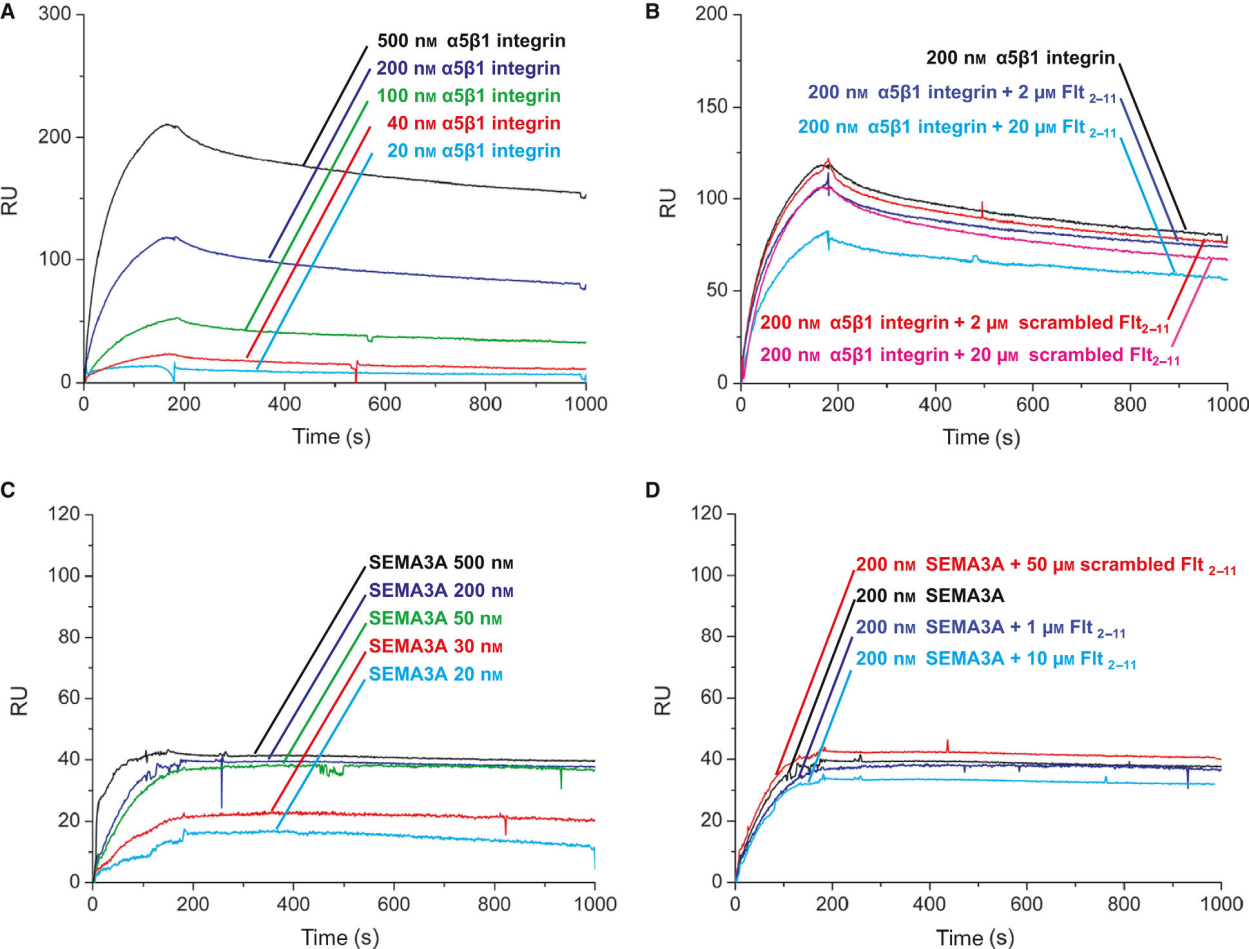
Flt_2‐11_ peptide does not interfere with NRP‐1 binding to either α5β1 integrin or semaphorin 3A. SPR analysis of NRP‐1 interaction with α5β1 integrin or semaphorin 3A, in the absence or presence of Flt_2‐11_ peptide or ‘scrambled’ Flt_2‐11_. NRP‐1 is immobilized on sensor chip. (A) α5β1 integrin is injected at different concentrations (from bottom to top: 20 nm, cyan; 40 nm, red; 100 nm, green; 200 nm, blue; and 500 nm, black). Kinetic and thermodynamic parameters are: *k*_on_ = 2.1 × 10^4^ M^−1^·s^−1^; *k*_off_ = 2.9 × 10^−4^ s^−1^; *K*_D_ = 14 ± 4 nm. (B) α5β1 integrin is injected at 200 nm concentration together with either Flt_2‐11_ peptide or scrambled Flt_2‐11_ peptide at different concentrations (no peptides, black; Flt_2‐11_ peptide 2 M μ and 20 μm, blue and cyan, respectively; scrambled Flt_2‐11_ peptide at 2 μm and 20 μm, red and purple, respectively). (C) Semaphorin 3A is injected at different concentrations (from bottom to top: 20 nm, cyan; 30 nm, red; 50 nm, green; 200 nm, blue; and 500 nm, black). Interaction parameters: *k*_on_ = 3.7 × 10^3^ M^−1^·s^−1^; *k*_off_ = 9 × 10^−5^ s^−1^; *K*_D_ = 24 ± 6 nm. (D) Semaphorin 3A is injected at 200 nm, and peptide Flt_2‐11_ at different concentrations (no peptides, black; Flt_2‐11_ peptide 1 μm and 10 μm blue and cyan, respectively; scrambled Flt_2‐11_ peptide 50 μm, red).

NRP‐1 binding by Flt_2‐11_ peptide was specific, since none of the four additionally tested peptides was able to bind either NRP‐1 (Fig. 1B) or NRP‐2 (data not shown). These four peptides were chosen because they all map on the surface of the second Ig‐like domain of VEGFR‐1 and are able to inhibit EC adhesion to sVEGFR‐1 [34, 36],

Since the Flt_2‐11_ peptide corresponds to a sequence within VEGFR‐1 second Ig‐like domain, we performed surface plasmon resonance (SPR) analyses to investigate whether Flt_2‐11_ peptide inhibited NRP‐1 interaction with VEGFR‐1. We found that Flt_2‐11_ peptide inhibited NRP‐1 binding to VEGFR‐1 with high efficiency (*K*_i_ = 20 nm; Fig. 2A,B). Inhibition of NRP‐1/VEGFR‐1 interaction by Flt_2‐11_ peptide was also specific, given that a ‘scrambled’ Flt_2‐11_ peptide, containing the same residues as Flt_2‐11_ but in a different order, had no effect on NRP‐1/VEGFR‐1 interaction (Fig. 2C). Conversely, Flt_2‐11_ peptide did not detectably affect NRP‐1 interaction with either α5β1 integrin (Fig. 3A,B) or semaphorin 3A (Fig. 3C,D).

### NRP‐1 is required for EC adhesion to sVEGFR‐1

**Fig. 4:**
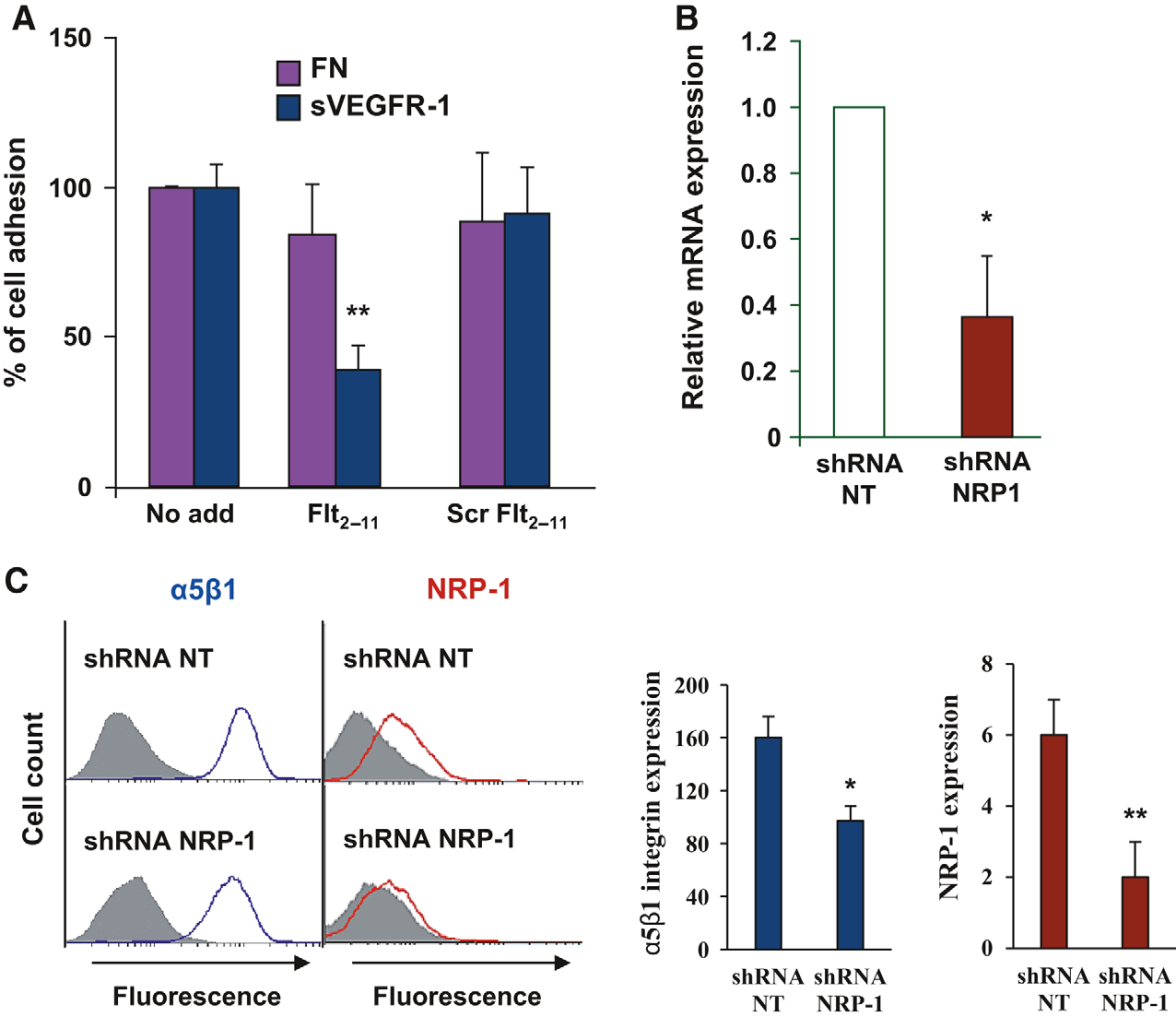
Evaluation of RNA interference for NRP‐1 in HUVECs. (A) EC adhesion to either sVEGFR‐1 or fibronectin (FN) in the presence of Flt_2‐11_ peptide or scrambled (Scr) Flt_2‐11_ peptide or in the absence of any peptide (No add). Results are expressed as percentage of basal EC adhesion to each protein with respect to the control (No add). Student’s *t*‐test: **P < 0.01. (B) Real‐time RT‐PCR on total RNA extracted from ECs, either interfered for NRP‐1 expression (shRNA NRP‐1) or transfected with a shRNA for an unrelated, not‐targeted gene (shRNA NT). Results are reported as relative fold expression with respect to shRNA NT‐transfected cells and represent the mean ± SE of experiments performed in triplicate out of three independent determinations. (C) FACS analysis was performed to determine integrin α5β1 and NRP‐1 levels in ECs interfered for NRP‐1 expression (shRNA NRP‐1) and transfected with a control shRNA (shRNA NT). Panels show the results of a representative experiment, and histograms indicate the quantification of α5β1 and NRP‐1 expression, evaluated as variations in the geometric mean fluorescence intensity. Results represent the mean values ± SE of four independent determinations. In B and C, Student’s *t*‐test: *P ≤ 0.05, **P ≤ 0.01.

We had previously reported that Flt_2‐11_ peptide specifically interfered with EC adhesion to sVEGFR‐1 but had no effect on EC adhesion to fibronectin, even if EC binding to both these extracellular matrix proteins is mediated by α5β1 integrin [34]. We show here that this effect is specific, since the scrambled Flt_2‐11_ peptide does not affect EC adhesion on either sVEGFR‐1 or fibronectin (Fig. 4A).

Taken together, the ability of Flt_2‐11_ peptide to (a) bind NRP‐1 and inhibit NRP‐1 interaction with VEGFR‐1 reported above, and (b) specifically inhibit EC adhesion to sVEGFR‐1, suggest that NRP‐1 interaction with VEGFR‐1 is required for ECs adhesion to sVEGFR‐1 to occur.

To test this hypothesis, we silenced NRP‐1 in ECs by small hairpin RNA (shRNA) interference.

We found, by real‐time RT‐PCR, that shRNA treatment reduced NRP‐1 mRNA levels by about 60% (Fig. 4B). Additionally, FACS analysis showed that shRNA treatment strongly reduced NRP‐1 protein expression, with respect to the levels observed in cells treated with control shRNA (Fig. 4C). Since NRP‐1 was previously shown to be involved in the regulation of α5β1 integrin trafficking [24], we evaluated integrin expression in our interfered cells. As shown in Fig. 4C, NRP‐1 mRNA interference slightly reduced α5β1 integrin membrane levels (by about 1/3).

**Fig. 5:**
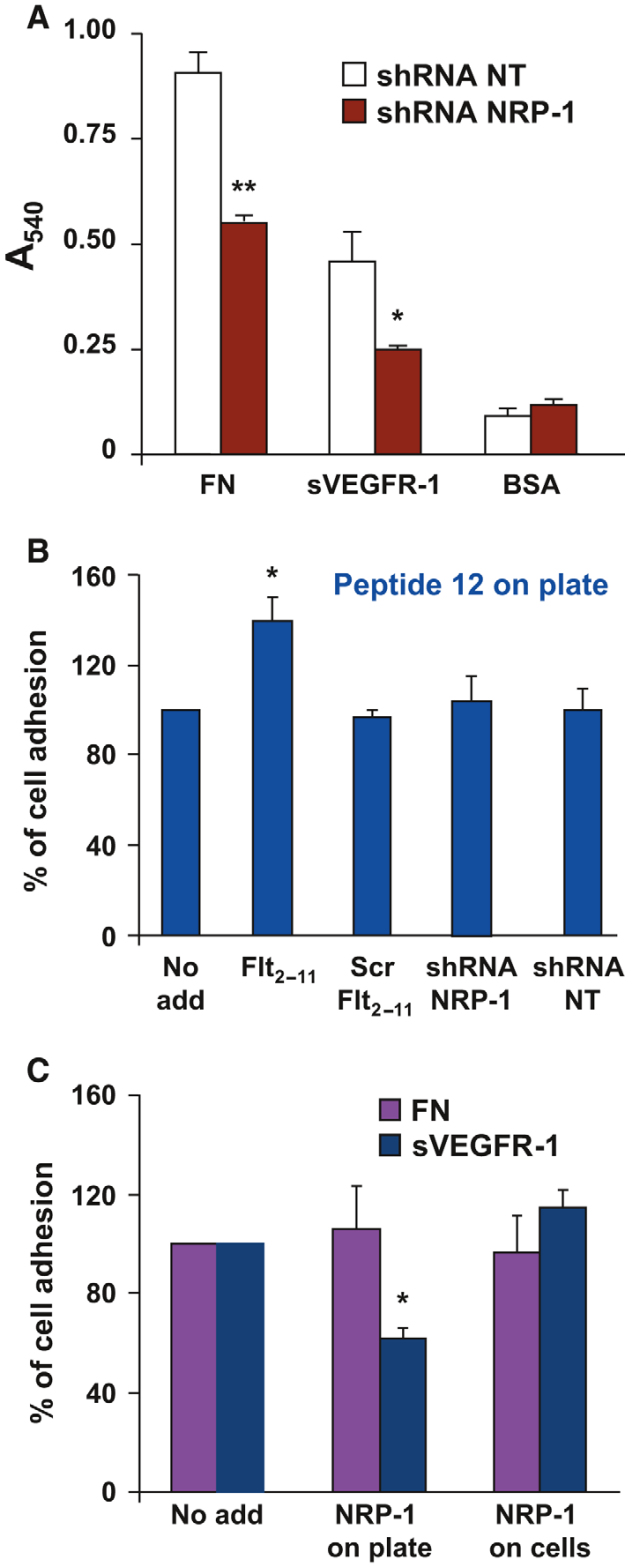
NRP‐1 interaction with sVEGFR‐1 is required for EC adhesion to sVEGFR‐1. (A) Adhesion of ECs, interfered for expression of NRP‐1 (shRNA NRP‐1), or of an unrelated, not‐targeted gene (shRNA NT), to sVEGFR‐1 or FN. Representative experiments performed in triplicate are shown; data are reported as mean ± SE. Student’s t‐test: *P ≤ 0.05; **P ≤ 0.01. Experiments were repeated at least three times with comparable results. (B) Adhesion of normal ECs or of ECs interfered for expression of NRP‐1 (shRNA NRP‐1) or of ECs interfered with an unrelated, not‐targeted gene (shRNA NT), to peptide 12, in the presence of Flt_2‐11_ peptide or Scr Flt_2‐11_ peptide, or in the absence of any peptide (No add). Results are expressed as percentage of basal EC adhesion to peptide 12. Student’s t‐test: *P ≤ 0.05. (C) EC adhesion to sVEGFR‐1 or FN, in the absence (No add) or in the presence of soluble NRP‐1, incubated with either the substrate‐coated plates (NRP‐1 on plate), or with ECs prior to their addition in the adhesion assay (NRP‐1 on cells). Results are expressed as percentage of basal EC adhesion on either sVEGFR‐1 or FN, with respect to the controls (No add). Representative experiments performed in triplicate are shown; data are reported as mean ± SE. Student’s t‐test: *P ≤ 0.05. Experiments were repeated at least three times with comparable results.

Using NRP‐1 interfered cells, we observed a reduction of EC adhesion both to fibronectin (47% reduction), in agreement with previously reported data [24], and to a greater extent, to sVEGFR‐1 (65% reduction) (Fig. 5A). These results indicate that NRP‐1 is required for effective EC binding to sVEGFR‐1, and the reduction of EC adhesion to sVEGFR‐1 is contributed by both an indirect role of NRP‐1, which is exerted through modulation of α5β1 integrin, and a direct role of NRP‐1 in EC adhesion to sVEGFR‐1. The reduction of EC adhesion to fibronectin, which is only determined by the indirect role of NRP‐1, is less pronounced (Fig. 5A).

### NRP‐1 interaction with the soluble VEGFR‐1 isoform is required for EC adhesion

To dissect whether interaction of NRP‐1 with the transmembrane or the soluble isoform of VEGFR‐1 was required for EC adhesion to sVEGFR‐1, we performed EC adhesion assays by substituting sVEGFR‐1 with a different adhesion substrate, named peptide 12. This is a seven amino acid‐long peptide that, like Flt_2‐11_, maps on the second VEGFR‐1 Ig‐like domain and that we previously reported to be able to directly bind α5β1 integrin and support EC adhesion, resulting into a pro‐angiogenic stimulus [34].

In this assay, no sVEGFR‐1 isoform is available, therefore tmVEGFR‐1 is the only VEGFR‐1 isoform that could interact with NRP‐1. As shown in Fig. 5B, addition of Flt_2‐11_ peptide does not reduce EC adhesion to peptide 12, indicating that this process does not require a putative NRP‐1 interaction with tmVEGFR‐1. Additionally, EC adhesion to peptide 12 is not reduced by downregulation of NRP‐1 expression by shRNA interference, indicating that this process does not require the presence of NRP‐1 (Fig. 5B). Interestingly, cell adhesion to peptide 12 is increased in the presence of Flt_2‐11_ peptide (Fig. 5B).

To investigate the reasons underlying EC ability to adhere to peptide 12, but not to the whole sVEGFR‐1, in the absence of NRP‐1, we analysed the three‐dimensional (3D) structure of the second VEGFR‐1 Ig‐like domain, which has been experimentally determined by X‐ray crystallography in complex with VEGF‐A165 [38]. This 3D structure comprises the whole peptide 12 sequence. Visual inspection and solvent accessible surface area measurement (see Table 1) indicated that peptide 12 residues Tyr220, Leu221, His223 and Arg224, which we previously demonstrated to contribute the most to the interaction with α5β1 integrin [34], are buried within the second VEGFR‐1 Ig‐like domain. A conformational change of this domain, resulting in the exposure of these four residues, must therefore take place to allow α5β1 integrin interaction with, and EC adhesion to, sVEGFR‐1.

**Table 1:** Availability of peptide 12 residues for interaction. SASA (Å^2^) of peptide 12 residues in the isolated peptide 12 and in the context of the 3D structure of the second Ig‐like domain of VEGFR‐1 (PDB identifier: 1FLT, Resolution: 1.7 Å). a.a.: amino acid type, three‐letter code; Nb: residue number in the PDB file; chain x, chain y: name of the chains of the two sVEGFR‐1 Ig‐like domain 2 copies present in the experimental structure; Peptide, Domain and Diff: SASA of each residue and of the whole peptide 12 in the free state and in the context of the whole domain, and difference between these values, respectively. Residues previously shown to play an essential role in EC adhesion and/or α5β1 integrin binding to sVEGFR‐1 [34] are bold.

a.a.	Nb.	Chain X			Chain Y		
		Peptide	Domain	Diff	Peptide	Domain	Diff
ASN	219	185.72	25.56	160.16	187.86	31.39	156.47
**TYR**	**220**	**216.75**	**4.15**	**212.6**	**221.19**	**5.36**	**215.83**
**LEU**	**221**	**175.74**	**64.17**	**111.57**	**169.71**	**66.21**	**103.5**
THR	222	135.71	6.5	129.21	131.26	6.18	125.08
**HIS**	**223**	**155.42**	**83.65**	**71.77**	**165.92**	**87.12**	**78.8**
**ARG**	**224**	**230.88**	**124.41**	**106.47**	**211.21**	**117.66**	**93.55**
GLN	225	211.59	132.04	79.55	209.21	202.16	7.05
Peptide 12	219–225	1311.81	440.48	871.33	1296.36	516.08	780.28

EC adhesion assays to sVEGFR‐1 or fibronectin in the presence of soluble NRP‐1 were also performed to further define the role of this molecule. As shown in Fig. 5C, when soluble NRP‐1 was added to the plates coated with either sVEGFR‐1 or fibronectin prior to EC addition, EC adhesion to sVEGFR‐1, but not to fibronectin, was impaired. These results indicate that soluble NRP‐1, bound to coated sVEGFR‐1, competes with the interaction between coated sVEGFR‐1 and NRP‐1 present on the EC membrane. On the other hand, when ECs were pretreated with soluble NRP‐1, allowing it to bind to the tmVEGFR‐1 isoform on ECs, cell adhesion to either sVEGFR‐1 or fibronectin was not detectably altered (Fig. 5C).

Taken together, these data indicate that the interaction between NRP‐1 present on ECs, and sVEGFR‐1 isoform in the extracellular matrix, supports sVEGFR‐1 interaction with α5β1 integrin and EC adhesion to sVEGFR‐1. Conversely, NRP‐1 interaction with tmVEGFR‐1 does not appear to play a role in EC adhesion to sVEGFR‐1.

### A five‐residue‐long peptide is sufficient to impair EC adhesion and migration

**Fig. 6:**
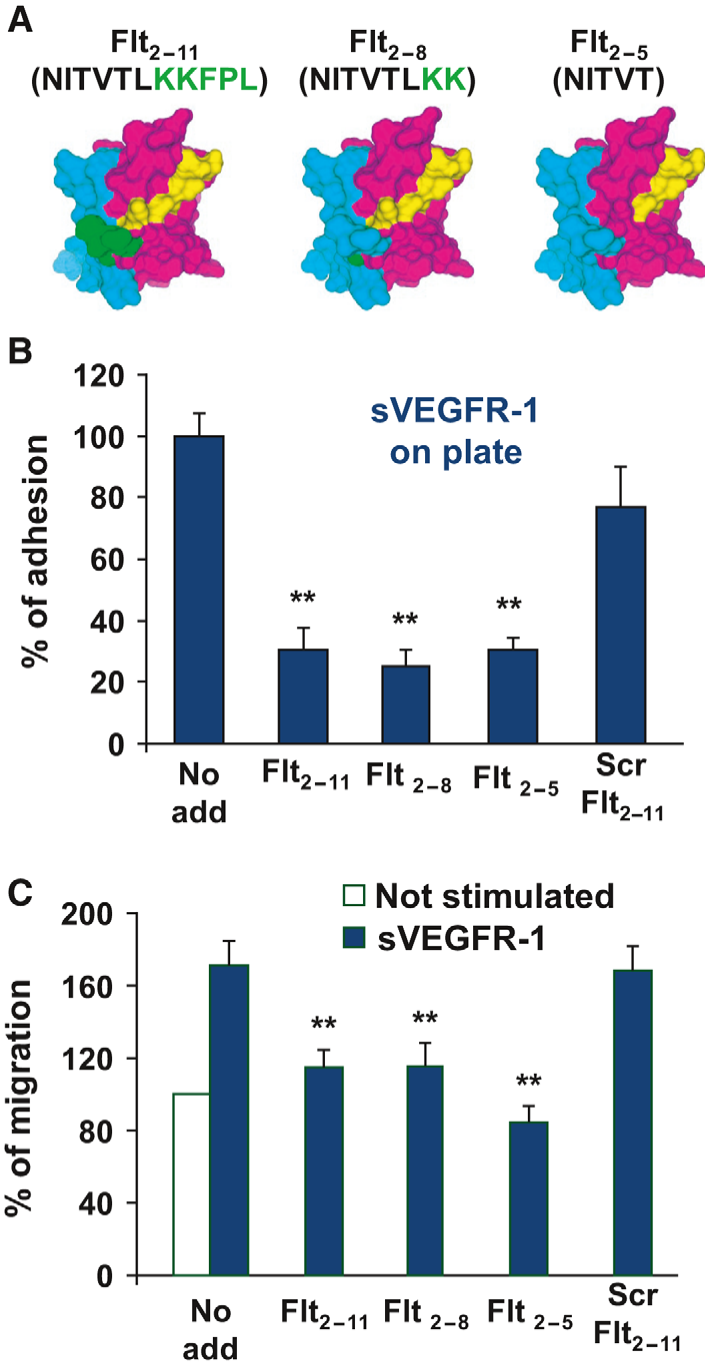
Effect of Flt_2‐8_ and Flt_2‐5_ peptides on EC adhesion and migration. (A) SASA of the 3D structure of VEGFR‐1 Ig‐like domain II. Colour coding: green, residues comprised in the Flt_2‐11_ or Flt_2‐8_ peptide that are in contact with VEGF‐A165; yellow, residues comprised in the Flt_2‐11_, Flt_2‐8_ or Flt_2‐5_ peptide that are not in contact with VEGF‐A165; cyan and magenta, other VEGFR‐1 residues that are and are not in contact with VEGF‐A165, respectively. The images were generated using the insightii program (Accelrys Inc.). (B) EC adhesion to sVEGFR‐1 in the presence of peptide Flt_2‐11_, Flt_2‐8_, Flt_2‐5_ or scrambled (Scr) Flt_2‐11_, or in the absence of any peptide (No add). Results are expressed as percentage of basal EC adhesion to sVEGFR‐1. (C) Migration towards sVEGFR‐1 in the presence of peptide Flt_2‐11_, Flt_2‐8_, Flt_2‐5_ or Scr Flt_2‐11_, or in the absence of any peptide (No add). Results are expressed as percentage of basal EC migration in the absence of any stimulus. In B and C, representative experiments performed in triplicate are shown; data are reported as mean ± SE. Student’s *t*‐test: **P ≤ 0.01, comparing peptide treated to untreated (No add) controls. Experiments were repeated at least three times with comparable results.

To identify the shortest peptide sequence able to interfere with EC adhesion to and migration towards sVEGFR‐1, we inspected the 3D structure of the second Ig‐like domain of VEGFR‐1 in complex with VEGF‐A165, which has been experimentally determined by X‐ray crystallography. Since we found that part of the region comprised within the Flt_2‐11_ peptide is involved in VEGF‐A165 binding (Fig. 6A), we designed two additional peptides, eight and five‐residue‐long, respectively, by eliminating three or all the five Flt_2‐11_ peptide residues located in the VEGF‐A165 binding region (Fig. 6A). Both Flt_2‐11_ derived peptides (*i.e*., Flt_2‐8_ and Flt_2‐5_) were able to significantly inhibit EC adhesion to (Fig. 6B), and migration towards, sVEGFR‐1 (Fig. 6C), to a similar extent as the Flt_2‐11_ peptide.

### NRP‐1 affinity for either sVEGFR‐1 or α5β1 integrin is higher than the affinity between sVEGFR‐1 and α5β1 integrin

The above‐reported results demonstrate that NRP‐1 binding to sVEGFR‐1 is required for EC adhesion to sVEGFR‐1, a process that we have previously demonstrated to be mediated by a direct interaction between sVEGFR‐1 deposited in the extracellular matrix and α5β1 integrin on EC membranes [31].

**Fig. 7:**
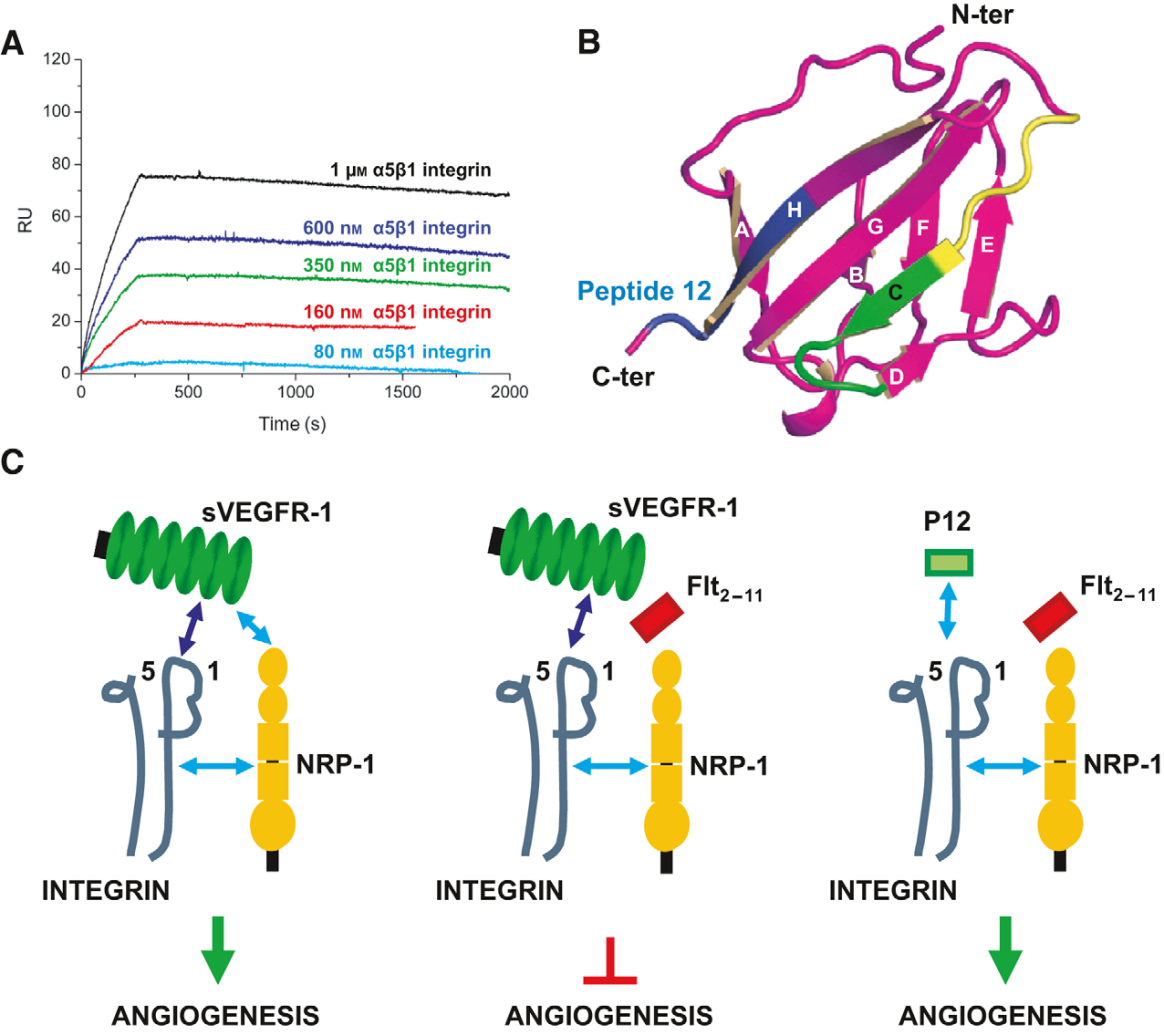
sVEGFR‐1/α5β1 integrin interaction. (A) SPR analysis of the interaction between sVEGFR‐1 and α5β1 integrin. sVEGFR‐1 is immobilized on sensor chips and α5β1 integrin is injected at different concentrations (from bottom to top: 80 nm, cyan; 160 nm, red; 350 nm, green; 600 nm, blue; and 1 μm, black). Interaction parameters: *k*_on_ = 1.5 × 10^3^ M^−1^·s^−1^; *k*_off_ = 2.9 × 10^−4^ s^−1^; *K*_D_ = 195 ± 40 nm. (B) Secondary structure representation of the 3D structure of VEGFR‐1 Ig‐like domain II. β‐strands are represented by arrows and labelled from A to H; loops are shown as tubular ribbons; the N‐ and C‐terminal ends of the domain are indicated by labels. Colour coding: peptide 12 (interacting with α5β1 integrin) is blue; Flt_2‐11_ peptide (interacting with NRP‐1) is yellow in the five‐residue NITVT region corresponding to the Flt_2‐5_ peptide (see text), and green in the remaining six‐residues region; the rest of the domain is magenta. (C) Proposed mechanism of action of Flt_2‐11_ peptide. Left: Light blue arrows indicate the high‐affinity interactions: (i) between NRP‐1 (yellow) and α5β1 integrin (black) on the EC membrane; and (ii) between NRP‐1 on the EC membrane and the sVEGFR‐1 substrate (green). The dark blue arrow indicates the low‐affinity interaction between α5β1 integrin on the EC membrane and the sVEGFR‐1 substrate. The low‐affinity interaction is stabilized by the two high‐affinity interactions, and the angiogenic stimulus is triggered. Centre: The Flt_2‐11_ peptide inhibits the high‐affinity interaction between NRP‐1 on the EC membrane and the sVEGFR‐1 substrate. The low‐affinity interaction between α5β1 integrin on ECs and the sVEGFR‐1 substrate is not sufficiently stable to trigger the angiogenic stimulus that starts upon integrin engagement. Right: The affinity of the interaction between α5β1 integrin (black) on the EC membrane and the P12 peptide substrate (light blue arrow) is high enough that additional stabilizing interactions are not required. Consequently, the α5β1 integrin/P12 peptide interaction takes place both in the presence and in the absence of the Flt_2‐11_ peptide.

To investigate the interplay between these molecules, we compared the affinities measured for each pairwise interaction with one another. Interestingly, the interaction between sVEGFR‐1 and α5β1 integrin measured by SPR is one order of magnitude weaker (*K*_D_ = 195 ± 40 nm; Fig. 7A) than NRP‐1 interaction with either sVEGFR‐1 (*K*_D_ = 25 ± 4 nm; Fig. 2A) or α5β1 integrin (*K*_D_ = 14 ± 4 nm; Fig. 3A), whereas the strength of the latter interactions is comparable. These results indicate that, if a ternary complex was formed among these three molecules, NRP‐1 interactions with sVEGFR‐1 and α5β1 integrin would have a stabilizing effect on sVEGFR‐1 interaction with α5β1 integrin.

To explore the possibility that a ternary complex among NRP‐1, sVEGFR‐1 and α5β1 integrin is formed, we inspected the primary and tertiary structure of VEGFR‐1 Ig‐like domain II, whose 3D structure has been experimentally determined by X‐ray crystallography in complex with VEGF‐A165 (Fig. 7B). Residues belonging to Flt_2‐11_ peptide, which interacts with NRP‐1, and peptide 12, which interacts with α5β1 integrin, correspond to separate regions of both VEGFR‐1 sequence (residues 164–174 and 219–225, respectively) and 3D structure (they partially overlap with β‐strands C and H of Ig‐like domain II, respectively) (Fig. 7B). In particular, the five‐residue‐long region of Flt_2‐11_ peptide, which is sufficient to inhibit EC adhesion and migration (Fig. 6A), maps on a sVEGFR‐1 Ig‐like domain II region opposite to that of peptide 12 (Fig. 7B). Thus, while neither α5β1 integrin nor NRP‐1 regions involved in the interaction with sVEGFR‐1, or with each other, are known, formation of a complex among NRP‐1, sVEGFR‐1 and α5β1 integrin cannot be excluded.

Figure 7C reports a schematic representation of the role of NRP‐1 in EC adhesion to sVEGFR‐1, and of the mechanism of action of the Flt_2‐11_ peptide, which have been proposed based on the results obtained in this work.

## Discussion

In this study, we analyse the role played by NRP‐1 in angiogenesis, by taking advantage of two previously reported peptides, both of which map on the extracellular region of VEGFR‐1 receptor.

First, we investigate in detail the mechanism underlying the anti‐angiogenic activity of the Flt_2‐11_ peptide, the name of which indicates that it comprises eleven residues mapping on the second Ig‐like domain of VEGFR‐1 [35]. Flt_2‐11_ peptide had been originally reported to exert an anti‐angiogenic activity, but the mechanism involved in this activity was not understood. However, Flt_2‐11_ peptide was demonstrated not to bind VEGF‐A165 or interfere with VEGF‐A165 binding to ECs [35]. Subsequently, we reported that Flt_2‐11_ peptide does not bind to α5β1 integrin [34]. In the present work, we show that the Flt_2‐11_ peptide specifically binds to NRP‐1 and disrupts NRP‐1 interaction with sVEGFR‐1. Additionally, we demonstrate that this peptide inhibits EC adhesion to, and migration towards, sVEGFR‐1, two processes mediated by the interaction of sVEGFR‐1 with α5β1 integrin. These results indicate that NRP‐1/sVEGFR‐1 interaction is required for a productive sVEGFR‐1/α5β1 integrin interaction to take place. Consequently, the previously reported anti‐angiogenic effect of the Flt_2‐11_ peptide is likely to be mediated by the peptide ability to interfere with sVEGFR‐1/NRP‐1 binding, which results in the inhibition of the pro‐angiogenic interaction between sVEGFR‐1 and α5β1 integrin.

The requirement of a sVEGFR‐1/NRP‐1 interaction for the efficient binding of sVEGFR‐1 to α5β1 integrin leads us to question the interplay among these three molecules during the EC adhesion process.

To answer this question, we take advantage of a different peptide previously studied by our group, named peptide 12, which also maps on the surface of VEGFR‐1 second Ig‐like domain, and mediates EC adhesion and α5β1 integrin binding [34]. In the present work, we show that ECs adhere to peptide 12 in the absence of NRP‐1, and that this adhesion is not affected by the Flt_2‐11_ peptide. These results imply that α5β1 integrin interaction with peptide 12 does not require NRP‐1 interaction with sVEGFR‐1. EC ability to adhere to peptide 12, but not to the whole sVEGFR‐1, in the absence of NRP‐1, can be explained based on the analysis of the experimentally determined 3D structure of VEGFR‐1 second Ig‐like domain. In this structure, the four residues previously demonstrated to be essential for the interaction with α5β1 integrin, namely Tyr220, Leu221, His223 and Arg224, are largely buried. To interact with α5β1 integrin, VEGFR‐1 needs to undergo a conformational change, leading to the exposure of these four residues. Therefore, the high‐affinity sVEGFR‐1/NRP‐1 interaction may contribute to stabilize a sVEGFR‐1 conformation where these residues are exposed, and available for the interaction with α5β1 integrin, thus facilitating EC adhesion on sVEGFR‐1. In agreement with this hypothesis, EC ability to adhere to peptide 12 in the absence of NRP‐1 may be ascribed to the fact that in peptide 12 the four aforementioned residues are fully available for the interaction with α5β1 integrin.

These observations also suggest that NRP‐1/sVEGFR‐1 interaction is not required to activate α5β1 integrin‐mediated signal transduction. As we have previously shown, EC adhesion to sVEGFR‐1 does not activate canonical α5β1 integrin signalling, such as phosphorylation of focal adhesion kinase or Shc, but acts through up‐regulation of myristoylated alanine‐rich C‐kinase substrate and persistent activation of Rac1, due to the involvement of radixin and Gα_13_ protein [32]. This special signalling pathway is also activated by EC adhesion to peptide 12 (data not shown), which is neither hampered by downregulation of NRP‐1 expression through shRNA interference, nor by inhibition of NRP‐1/sVEGFR‐1 interaction by Flt_2‐11_ peptide.

Surprisingly, in the absence of sVEGFR‐1 substrate, Flt_2‐11_ peptide increases cell adhesion to peptide 12. Based on the mechanism of action of Flt_2‐11_ peptide disclosed in this work, it is reasonable to speculate that Flt_2‐11_ peptide inhibits NRP‐1 interaction with the transmembrane form of VEGFR‐1, thus increasing the amount of NRP‐1 available to exert the α5β1 integrin cell adhesion‐enhancing function, as previously observed [24].

To investigate the relative strength of interaction of NRP‐1, sVEGFR‐1 and α5β1 integrin with one another, we perform *in vitro* SPR experiments. The results of these experiments indicate that the *K*_D_ of both interactions involving NRP‐1, namely NRP‐1/sVEGFR‐1 and NRP‐1/α5β1 integrin, are 10‐fold lower than that of the sVEGFR‐1/α5β1 integrin interaction. In case a ternary complex was formed among these molecules, NRP‐1 would have a stabilizing role on the sVEGFR‐1/α5β1 integrin interaction. Analysis of sVEGFR‐1 Ig‐like domain 2 sequence and structure indicates that Flt_2‐11_ peptide and peptide 12 residues, which bind NRP‐1 and α5β1 integrin, respectively, are not overlapping. Therefore, a simultaneous interaction involving all three molecules is possible. Indeed, NRP‐1 has been reported to be involved in ternary complexes with other macromolecules. As an example, NRP‐1 and plexin 1 form a complex whose affinity for semaphorin 3A is enhanced with respect to each component [41]. Additionally, NRP‐1 binds PDGF‐D and acts as a co‐receptor, enhancing the formation of a complex between the growth factor and PDGFRβ [42]. Further, β3 integrin negatively regulates VEGF‐A165‐mediated angiogenesis by limiting the interaction of NRP‐1 with VEGFR‐2 [43]. Unfortunately, our attempts at isolating a ternary complex formed by NRP‐1, sVEGFR‐1 and α5β1 integrin in ECs have not been successful so far.

Because of its peculiar mechanism of action, the Flt_2‐11_ peptide is an attractive lead for the development of anti‐angiogenic compounds for therapeutic applications. Indeed, most of the previously developed therapeutics for pathological angiogenesis target VEGF‐A165 interaction with transmembrane receptors and determine a reduction of VEGFR‐2‐mediated signalling [44, 45]. Small peptides that inhibit VEGF‐A165 binding to NRP‐1 hamper tumour growth and angiogenesis in breast cancer [46] and lung carcinoma [47] animal models. Anti‐NRP‐1 antibodies enhance the antitumor effects of anti‐VEGF‐A165 antibodies in a mouse xenograft model [48]. Inhibition of NRP‐1 interaction with placenta growth factor has antitumor effects in a mouse model of medulloblastoma [46]. In the developing retina, the combination of anti‐NRP‐1 and anti‐VEGF‐A165 antibodies results in enhanced blood vessel regression [48]. A peptide interfering with VEGF‐A165 binding to NRP‐1 suppresses angiogenesis in experimental arthritis [49]. Conversely, the anti‐angiogenic effect of the Flt_2‐11_ peptide is not mediated by inhibition of NRP‐1 binding to VEGF family members, and subsequent reduction of signalling mediated by transmembrane VEGFRs [35], but by a novel mechanism, consisting in the inhibition of NRP‐1/sVEGFR‐1 interaction. Therefore, compounds acting with the same mechanism as Flt_2‐11_ peptide could be used as an alternative to classic anti‐angiogenic molecules or in synergy with them in combined, more effective protocols.

As a first step to develop such anti‐angiogenic agents, in this work we design shorter variants of Flt_2‐11_ peptide and test their biological activity. Analysis of the 3D structure of VEGFR‐1 second Ig‐like domain in complex with VEGF‐A165, which has been experimentally determined by X‐ray crystallography, revealed that some of the residues present in the Flt_2‐11_ peptide are involved in VEGF‐A165 binding. To further reduce any chance of interference with VEGF‐A165 activity, we designed two Flt_2‐11_ peptide variants comprising the first eight, and the first five, N‐terminal residues, and named them Flt_2‐8_ and Flt_2‐5_, respectively. Both peptides result to be able to impair α5β1 integrin‐mediated EC adhesion to, and migration towards, sVEGFR‐1 with an efficacy comparable to that of the parent eleven‐residue Flt_2‐11_ peptide. Consequently, both peptides, and especially the five‐residue Flt_2‐5_ peptide, represent novel potential angiogenesis inhibitors or lead compounds, based on which nonpeptide molecules acting with the same mechanism may be developed.

In this work, we also find that Flt_2‐11_ peptide directly interacts with NRP‐2. This protein has been previously shown to take part in a ternary complex with VEGFR‐1 and VEGF‐A165 [27]. However, NRP‐2 and VEGFR‐1 were not demonstrated to directly interact with each other. Interestingly, NRP‐2 has been shown to interact with α5β1 integrin and have a role in modulating cell/cell and cell/matrix adhesion [39, 40]. Further studies will be required to elucidate the biological role of the NRP‐2/VEGFR‐1 interaction, and whether the two molecules interact directly or through VEGF‐A165.

In summary, the results presented in this work demonstrate that the previously reported, direct NRP‐1/sVEGFR‐1 interaction is required for both EC adhesion to sVEGFR‐1 and EC migration towards sVEGFR‐1. Moreover, we discovered that the previously reported anti‐angiogenic Flt_2‐11_ peptide inhibits the NRP‐1/sVEGFR‐1 interaction. This led to the development of two shorter peptides, named Flt_2‐8_ and Flt_2‐5_, both of which hamper EC adhesion to, and migration towards, sVEGFR‐1 by inhibiting NRP‐1/sVEGFR‐1 interaction as well. This mechanism of action does not involve the inhibition of VEGF‐A165 binding to its transmembrane receptors. Therefore, the peptides studied in this work are expected to act synergistically with classic anti‐angiogenic agents and may be used in combination therapies for the treatment of tumours and other processes associated with pathological angiogenesis.

## Materials and methods

### Reagents

Recombinant human VEGFR‐1/Fc (321‐FL‐050), human NRP‐1 (3870‐N1) and human NRP‐2/Fc (2215‐N2) were purchased from R&D Systems (Minneapolis, MN, USA). Fibronectin (F1141) and gelatin (G1393) were obtained from Sigma‐Aldrich (St Louis, MO, USA). Purified α5β1 integrin (CC1052), octyl‐β‐d‐glucopyranoside formulation, was from Merck Millipore (Billerica, MA, USA). Peptide synthesis was carried out by Primm (Milan, Italy). Flt_2‐11_ peptide sequence was NITVTLKKFPL and scrambled Flt_2‐11_ peptide sequence was LVPLKIKNTFT.

### Cell culture

Human umbilical vein endothelial cells (HUVEC) were isolated from freshly delivered umbilical cords, as previously described [50], cultured in Endothelial Cell Growth Medium‐2 Kit from Clonetics (EGM‐2, CC4176, Lonza, Basel, Switzerland), and used up to passage 6. For functional assays, HUVEC were resuspended in Endothelial Basal Medium‐2 (EBM‐2, CC3156, Lonza). A pool of cells derived from four different individuals was used.

### Solid‐phase binding assays

Immunological 96‐multiwell plates were coated overnight at 4 °C with saturating amounts (2 µg·mL^−1^) of human NRP‐1, human NRP‐2/Fc, or human sVEGFR‐1/Fc, in 25 mm Tris/HCl, 150 mm NaCl, 1 mm MgCl_2_ (buffer A). Plates were then blocked for 2 h at room temperature with 1% BSA/PBS. Biotinylated Flt_2‐11_ peptide or other biotinylated sVEGFR‐1‐derived peptides [34] were then added to a final concentration of 500 µg·mL^−1^ for 1 h at room temperature. After three washes with buffer A, streptavidin‐alkaline phosphatase‐conjugated and the appropriate substrate (4‐nitrophenylphosphate, Roche Diagnostics, Basel, Switzerland) were then used for detection of bound peptides. Absorbance was determined at 405 nm, using a Microplate reader 3550‐UV (Bio‐Rad, Hercules, CA, USA). Experiments were performed in triplicate and repeated at least three times with comparable results.

### Cell adhesion assay

Solid support for adhesion assay was prepared by incubating immunological 96‐multiwell plates with 20 µg·mL^−1^ VEGFR‐1/Fc chimera, or fibronectin solubilized in PBS. After 2 h, the coating solution was removed, and the well surface was blocked with 3% BSA in PBS for 18 h, before plating ECs in serum‐free medium at 3 × 10^4^ cells per well. After incubation at 37 °C for 1 h, the wells were washed with PBS and attached cells were fixed with 3% formaldehyde and stained with 0.5% crystal violet. Attachment efficiency was determined by quantitative dye extraction and spectrophotometric measurement of the absorbance at 540 nm, using a Microplate reader 3550‐UV (Bio‐Rad). In competition experiments, peptides Flt_2‐11_, scrambled Flt_2‐11_, Flt_2‐8_ or Flt_2‐5_, at 20 µg·mL^−1^, were added during the adhesion assay. Adhesion to peptide 12 was carried out by using maleic anhydride activated microplates (Reacti‐Bind, Pierce, Rockford, IL, USA) coated with 500 µg·mL^−1^ of the peptide. Experiments were performed in triplicate and repeated at least three times with comparable results.

### RNA interference

MISSION Lentiviral Transduction Particle system was used (Sigma‐Aldrich). Subconfluent HUVEC were infected at a multiplicity of infection (MOI) = 1, with either NRP‐1 shRNA (SHCLNV‐NM 003873, clone TRCN0000300917, Sigma‐Aldrich) or an unrelated, not‐targeted non‐mammalian shRNA (Sigma‐Aldrich). After 24 h from infection, cell medium was changed with medium supplemented with 0.6 µm puromycin, to select cells infected with the virus containing the shRNA sequences.

After one week, interfered cells were used in cell adhesion assays, as described above. To evaluate levels of NRP‐1 mRNA expression in silenced cells, real‐time RT‐PCR was performed. Total RNA was extracted from interfered cells, and cDNA was obtained using Superscript III First‐Strand System (Invitrogen, Carlsbad, CA, USA), according to the manufacturer's protocol. Real‐time RT‐PCR was performed by the dual‐labelled fluorogenic probe method, using ABI Prism 7000 sequence detector (Perkin Elmer, Groningen, The Netherlands). Expression levels were calculated by the relative standard curve methods. Primers used were: NRP‐1 forward 5'‐GCCACAGTGGAACAGGTGAT‐3' and reverse 5'‐GGAAACTCTGATTGTATGGTGCTG‐3'; β‐actin forward 5'‐CATCGAGCACGGCATCGTCA‐3' and reverse 5'‐TAGCACAGCCTGGATAGCAAC‐3'.

### Flow cytometry analysis

Cells were harvested, allowed to recover for two hours in a rotating wheel at room temperature and washed with PBS. Aliquots of 5 × 10^5^ cells were then incubated on ice in 2% BSA/PBS for 30 min with the following mouse monoclonal antibodies. For integrin α5β1 labelling, we used 1 µg of anti‐α5β1 (MAB1969, Chemicon International, Temecula, CA, USA), washed with PBS and incubated with a secondary goat anti‐mouse IgG (Fc specific)‐FITC antibody (1 : 100, F2772, Sigma‐Aldrich) on ice in 2% BSA/PBS for 30 min. For NRP‐1 labelling, we used 10 µL of anti‐human NRP‐1‐PE (CD304‐PE, Miltenyi Biotec, Bergisch Gladbach, Germany) or mouse IgG1 control‐PE (Becton Dickinson, Franklin Lakes, NJ, USA). After washing with PBS, cells were analysed using a FACScan flow cytometer (Becton & Dickinson).

### Migration assays

Cell migration was analysed using Boyden chambers equipped with 8 µm pore diameter polycarbonate filters (Nuclepore, Whatman Incorporated, Clifton, NJ, USA). Human sVEGFR‐1 stimulus (5 µg·mL^−1^) for chemotaxis was added to the lower chamber and HUVEC (1.5 × 10^5^) were loaded into the upper chamber. To test the effect of peptides Flt_2‐11_, scrambled Flt_2‐11_, Flt_2‐8_ or Flt_2‐5_ on cell migration, cells were preincubated for 30 min at room temperature in migration medium (0.1% BSA in EBM‐2/heparin medium) alone (No add) or containing one of the peptides at 40 µm concentration. Cells were then loaded into the Boyden chambers without removing the peptides, and migration towards sVEGFR‐1 was analysed. After 2‐h incubation in a CO_2_ incubator at 37 °C, filters were removed, cells were fixed in 3% paraformaldehyde in PBS and stained in 0.5% crystal violet. Cells from the upper surface were removed by wiping with a cotton swab, and the chemotactic response was determined by counting the migrating cells attached to the lower surface of the filter in 12 randomly selected microscopic fields (×200 magnification) per experimental condition. Quantification was performed by two independent observers, blinded to the examined condition. Experiments were performed in triplicate and repeated at least three times with comparable results.

### SPR measurements

SPR experiments were carried out using a BIACORE X system (Biacore AB, Uppsala, Sweden). The sensor chips (CM5, Biacore AB) were chemically activated by injection of 35 µL of a 1 : 1 mixture of *N*‐hydroxysuccinimide (50 mm) and *N*‐ethyl‐*N’*‐(3‐dimethylaminopropyl)carbodiimide (200 mm) at a flow rate of 5 µL·min^−1^ [51]. For experiments where VEGFR‐1/Fc is the immobilized ligand, the procedure described in [52] was followed. Briefly, protein A was immobilized using amine coupling with *N*‐ethyl‐*N*’‐(3‐dimethylaminopropyl)carbodiimide hydrochloride and N‐hydroxysuccinimide to a density of 1000–2000 resonance units (RU). The remaining N‐hydroxysuccinimide ester groups were blocked by injecting 1 m ethanolamine hydrochloride (35 µL). Recombinant human VEGFR‐1/Fc was captured to approximately 200 RU. Interaction with analytes was achieved in HEPES‐buffered saline (10 mm HEPES, pH 7.4; 150 mm NaCl; 0.005% surfactant P20). For association measurements, the analyte was injected at a rate of 20 mL·min^−1^. Dissociation was obtained with HEPES‐buffered saline applied over the surface at 20 mL·min^−1^ for 30 min. Recombinant human NRP‐1 was immobilized on the activated sensor chip via amine coupling. The reaction was carried out in 20 mm sodium acetate at pH 5.0, which is about 2 points below the theoretical NRP‐1 isoelectric point calculated using the program pI‐tool [53]. The remaining N‐hydroxysuccinimide ester groups were blocked by injecting 1 m ethanolamine hydrochloride (35 µL). Interaction with the analyte was achieved in HEPES‐buffered saline. For association measurements, the soluble ligand was injected at a rate of 30 mL·min^−1^. Dissociation was obtained with HEPES‐buffered saline applied over the surface at 30 mL·min^−1^ for 30 min. In control experiments, the sensor chip was treated as described above in the absence of immobilized proteins.

RU express the mass concentration‐dependent changes in the observed SPR signal, that is, the refractive index on the sensor chip surface. Changes in this signal are determined by the interaction between the immobilized protein (i.e., either NRP‐1 or VEGFR‐1) with the analyte protein (i.e., VEGFR‐1/Fc, α5β1 integrin or semaphorin 3A). Typically, a response change of 1000 RU corresponds to a change in the surface concentration on the sensor chip of about 1 ng of protein per mm^2^. Global fitting analyses of association and dissociation data for all ligand concentrations were carried out using biaevaluation software 3.0 (Biacore, Uppsala, Sweden), which allows the ratio between association and dissociation constants (*k*_on_/*k*_off_) to be accurately determined. A simple 1 : 1 Langmuir model was employed to fit the data, where: A + B ↔ AB; A: analyte; B: ligand; AB: complex between A and B; *k*_on_: association rate constant (M^−1^·s^−1^); *k*_off_: dissociation rate constant (s^−1^). Scatchard analysis of *R*_eq_ dependence on analyte concentration was also performed to calculate the equilibrium dissociation constant.

### Sequence and structure analysis

The 3D structure of the second VEGFR‐1 Ig‐like domain in complex with VEGF [38] was downloaded from the Protein Data Bank (PDB: www.rcsb.org) [54] and analysed using insightii (Accelrys Inc.) and Swiss‐PDBViewer [55] (http://www.expasy.org/spdbv/). Solvent accessible surface area (SASA) was calculated using Naccess (http://wolf.bms.umist.ac.uk/naccess/).

### Statistical analysis

Statistical significance of the differences between pairs of groups was assessed by two‐tailed Student’s *t*‐test. Differences were statistically significant when *P* < 0.05.

## Conflict of interest

The authors declare that they have no conflicts of interest with the contents of this article. The sources of financial support for the conduct of the research and preparation of the article did not perform any role in study design; in the collection, analysis and interpretation of data; in the writing of the report and in the decision to submit the article for publication.

## Author contributions

GC designed SPR experiments, performed part of them, analysed results and wrote the paper. CMF designed cell adhesion experiments, performed part of them, analysed results and wrote the paper. PML designed cell migration experiments, analysed results and revised the paper. MU performed part of the SPR experiments. FR performed cell migration experiments. PDM performed part of the Bioinformatics analyses. AO performed part of the cell adhesion experiments. VM designed Bioinformatics analyses, performed part of them, analysed results and wrote the paper.

### Peer Review

The peer review history for this article is available at https://publons.com/publon/10.1111/febs.16119.
